# Evaluation of the Adequacy of Using the Supraglottic Airway Device (i-gel®) in Cases With Epiglottic Masses: A Mannequin Simulation Study

**DOI:** 10.7759/cureus.74734

**Published:** 2024-11-29

**Authors:** Tomoyuki Matsumoto, Tatsushige Iwamoto, Yasufumi Nakajima, Kei Houri, Takatoshi Tsujimoto, Hiroatsu Sakamoto, Atsuhiro Kitaura, Yoshinobu Nakayama

**Affiliations:** 1 Anesthesiology and Critical Care, Kindai University Faculty of Medicine, Osaka, JPN; 2 Anesthesiology and Perioperative Medicine, Kindai University Faculty of Medicine, Osaka, JPN

**Keywords:** epiglottic tumor, mannequin, simulation, supraglottic airway device, ventilation

## Abstract

Background: Epiglottic masses are often asymptomatic, making them difficult to detect during preoperative examinations. Consequently, anesthesiologists may face ventilation difficulties with no apparent cause. Epiglottic masses can sometimes obstruct laryngoscope insertion into the epiglottic vallecula, complicating general anesthesia induction. In such cases, supraglottic airway insertion may be a viable alternative; however, the limited case reports on its use for epiglottic masses make its applicability unclear. Therefore, we test the hypothesis that a larger laryngeal artificial mass could obstruct the view of the larynx, even when supraglottic airways are used in a mannequin study.

Methods: We utilized an airway management simulator (Air Sim Multi®: Nihon 3B Scientific, Japan) to place various sizes of artificial masses (tumors) above the epiglottis. The groups included a control group with no mass, small size mass group, middle size mass group, and large size mass group. The supraglottic airway (i-gel®: Intersurgical, UK) was then inserted 10 times. We categorized the view of the vocal cords using a bronchoscope inserted through the tip of the cuff according to the Cormack-Lehane classification. In addition, we performed pressure-controlled ventilation, adjusting the inspiratory pressure from 10 cm H₂O to 25 cm H₂O, while measuring the tidal volumes.

Results: The Cormack-Lehane classification grade increased in correlation with the mass size. In each inspiratory pressure, tidal volume decreased in correlation with the mass size. Furthermore, in the large-size mass group, even at an inspiratory pressure of 25 cm H₂O, achieving the tidal volume required for general adult respiratory management was deemed difficult.

Conclusion: In a mannequin study, we observed that epiglottic masses significantly increased the Cormack-Lehane classification grade and reduced tidal volume, with these effects correlating with the size of the mass. This finding suggests that the appropriateness of using a supraglottic airway may depend on the size and weight of the epiglottic mass.

## Introduction

The mass of the epiglottis may be cystic, granulomatous, infectious, neoplastic, or manifestations of a systemic disease. They can vary in size and may be solitary or multiple. The exact causes of epiglottic masses vary in etiology but are generally thought to result from obstruction of mucous glands, prior infections, inflammatory processes, and direct throat injuries [1]. When epiglottic masses become larger or infected, they may cause various symptoms, including dysphagia, odynophagia, sore throat, and airway compromise. Epiglottic masses that are symptomatic or cause airway obstruction may require surgical excision. However, they are generally asymptomatic, making them hard to detect during preoperative examinations [2]. As a result, anesthesiologists may encounter unexpected ventilation challenges without obvious or identifiable causes.

The presence of epiglottic mass can sometimes hinder the insertion of the laryngoscope into the epiglottic vallecula, potentially obstructing the induction of general anesthesia [2,3]. In such cases, supraglottic airway insertion may be a viable alternative. Supraglottic airway devices are commonly recommended for securing the airway during general anesthesia, including in difficult airway management, according to the guideline [4]. They are designed to be placed above the vocal cords to maintain an open airway and facilitate ventilation without endotracheal intubation. Furthermore, the combination of supraglottic airway devices and endotracheal fiber optics enables endotracheal intubation [5]. However, its effectiveness for airway management in cases involving epiglottic masses remains uncertain.

In the present study, we tested the hypothesis that a larger laryngeal artificial mass would obstruct the view of the larynx, even when using supraglottic airways, and evaluated the primary outcome of the Cormack-Lehane classification grade assigned during the procedure. In addition, we evaluated the secondary outcome that changes in tidal volume would correlate with the diameter of the mass in a mannequin simulation study.

## Materials and methods

**Figure 1:**
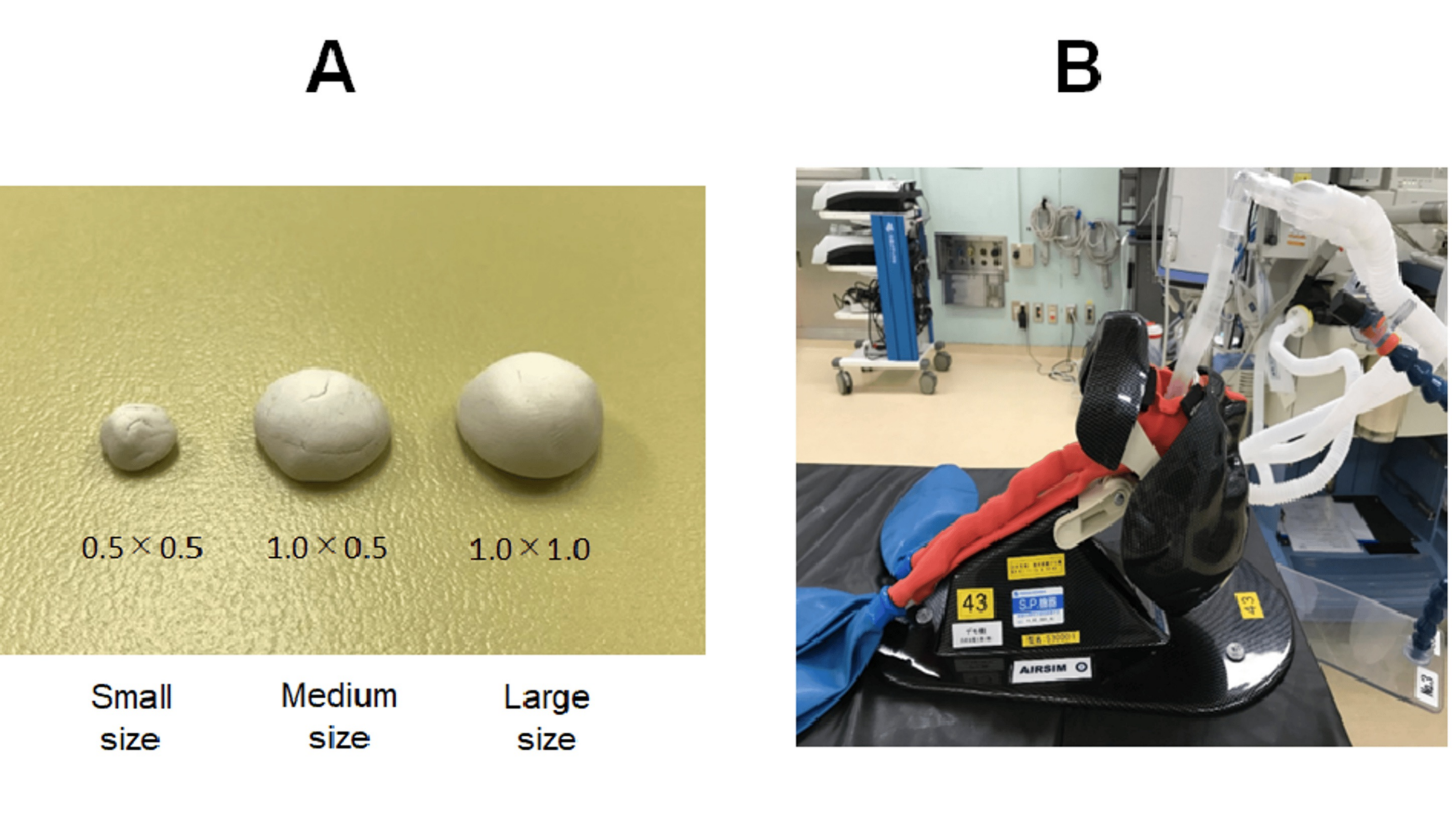
Artificial tumor (mass) size and airway management simulator A. Artificial tumor (mass) model, B. Airway management simulator

No ethics approval was required for conducting this study. We utilized an airway management simulator (Air Sim Multi®: Nihon 3B Scientific, Japan) by putting various sizes of mass above the epiglottis and inserting the supraglottic airway device i-gel® (Intersurgical, UK). A hemispherical mass model was created and placed in the lingual (anterior) portion of the epiglottis of an airway management simulator. The sizes of the artificial mass were as follows (radius × height (cm)): control group with no mass, small-size group with a mass of 0.5 × 0.5 cm (0.5 g), middle-size group with a mass of 1.0 × 0.5 cm (1.5 g), and large-size group with a mass of 1.0 × 1.0 cm (3 g). The sizes were based on previous studies of laryngeal masses, which showed that 5% of epiglottic cysts are ductal cysts, typically less than 1 cm in size, although they may occasionally grow larger (Figure 1) [6,7].

An i-gel® size 3 was inserted 10 times in each group, and the degree of glottic obstruction caused by the mass model was observed using a bronchoscope inserted through the tip of the cuff. We categorized the view of the larynx between grade I (the glottis (vocal cords) is fully visible), grade II (only part of the glottis is visible, with the posterior portion in view but not the anterior), grade III (only the epiglottis is visible, with no part of the glottis visible), and grade IV (neither the epiglottis nor the glottis is visible; only the tongue is seen) based on the Cormack-Lehane classification grades. In addition, pressure-controlled ventilation was performed, changing the inspiratory pressure from 10 cmH₂O to 25 cmH₂O in 5 cmH₂O increments, and tidal volumes were measured by an Apollo anesthesia machine (Drager, Germany). The tidal volume was measured every 10 supraglottic airway insertions, and the average value was calculated.

Statistics

A two-way ANOVA with repeated measurements was performed to compare tidal volumes between groups at each inspiratory pressure, followed by Bonferroni post-hoc tests. A p-value < 0.05 was considered statistically significant.

## Results

Image of the degree of glottic obstruction observed with a bronchoscope from the cuff of the i-gel®

**Figure 2:**
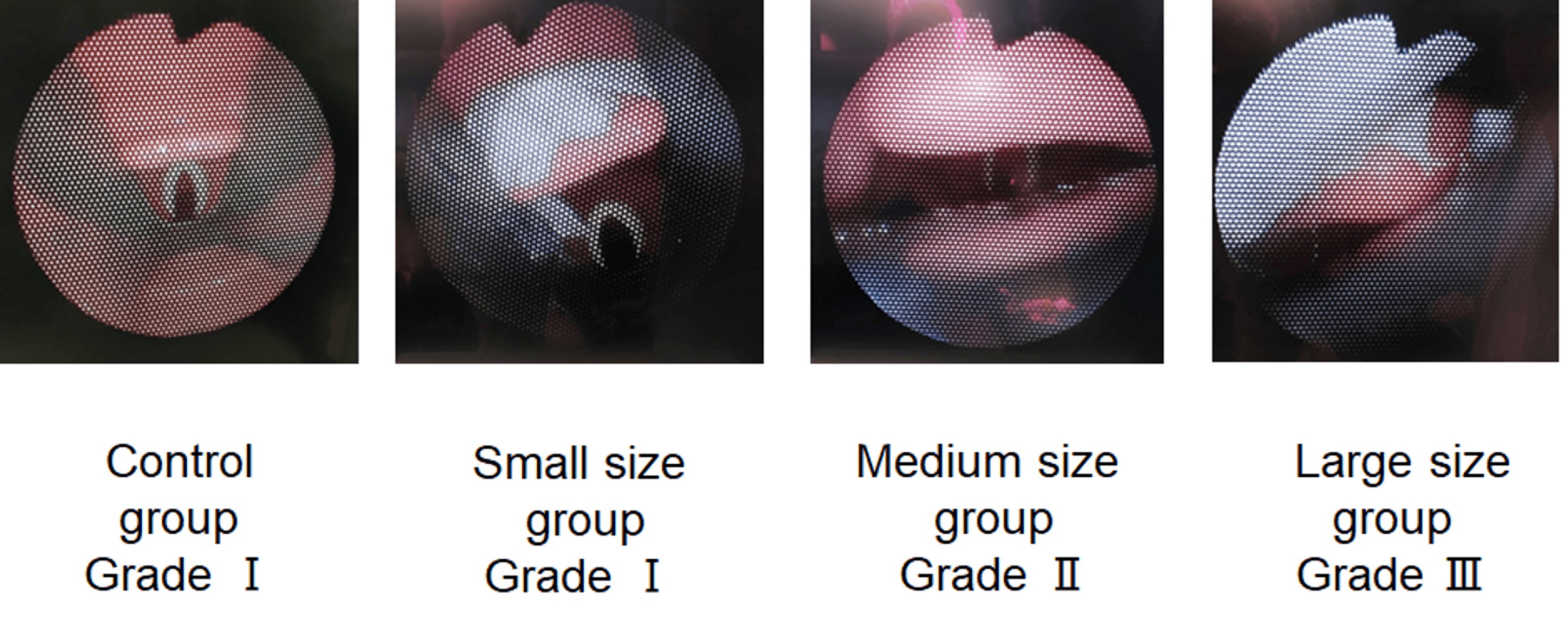
Cormack–Lehane classification grade according to the view of the vocal cords observed using a bronchoscope inserted through the tip of the cuff and the typical view of the larynx in each group. The Cormack–Lehane classification grade increased in correlation with the mass size.

The appearance of the larynx as viewed under bronchoscopy is shown. As demonstrated, the presence of the mass above the epiglottis caused the epiglottis to be pushed downward. Consequently, the Cormack-Lehane classification grade increased in correlation with mass size, although the grade did not differ across 10 trials of supraglottic insertion (Figure 2).

Average tidal volume

**Figure 3:**
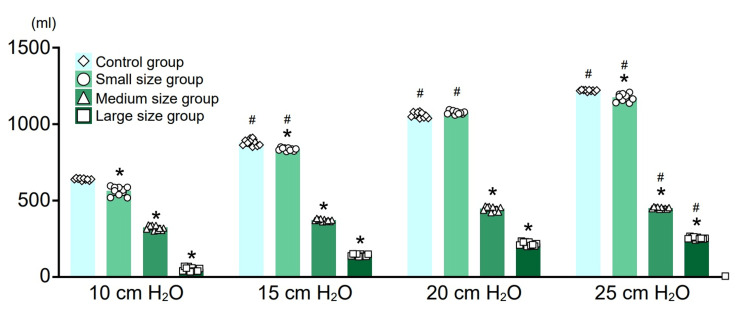
Tidal volume measurement in each group with inspiratory pressure changes from 10 to 25 cmH₂O in 5 cmH₂O increments. At the same inspiratory pressure, the tidal volume decreased in correlation with the mass size. * P < 0.05 with the control group at the same inspiratory pressure.＃P < 0.05 with the same size mass group at the inspiratory pressure of 10 cmH₂0.

In all mass groups, the tidal volume increased in correlation with the inspiratory pressure. In addition, at the same inspiratory pressure, tidal volume decreased in correlation with mass size. Particularly, in the large-size mass group, even when the inspiratory pressure was set to 25 cmH₂O, the tidal volume obtained was only about 200 ml (Figure 3).

## Discussion

Laryngeal masses can occur throughout the larynx but are commonly found in supraglottic locations, such as the epiglottis and vallecula [1]. Supraglottic airways, including those used in prehospital and cardiac arrest settings, offer a secure alternative to endotracheal intubation. However, they may not be suitable in cases of poor mouth opening, pharyngeal pathology, or obstruction at or below the level of the larynx. Despite their widespread use, the definitive role of the supraglottic airways has yet to be clearly established [8]. Epiglottic masses are often asymptomatic, making detection difficult during preoperative assessments. As a result, anesthesiologists may face unexpected ventilation issues, and supraglottic airways may be needed to secure the airway. We found that it might be a useful option in difficult airway management for small epiglottic masses; however, in cases of large masses, they may obscure the airway opening. In this study, the tidal volume decreased significantly in correlation with the mass size in a mannequin study. Furthermore, in the large-size group, even general adult respiratory management was deemed difficult.

The findings of the current study can be explained by the folding of the epiglottis, which occurred as a result of the weight and mass of the laryngeal growth. This mechanical deformation of the epiglottis led to significant airway obstruction, restricting airflow and causing increased resistance. As a consequence of this obstruction, the Cormack-Lehane classification grade was elevated, indicating a more difficult intubation scenario, likely due to the limited visibility of the glottic opening. Furthermore, the airway obstruction resulted in a noticeable decrease in tidal volume during pressure-controlled ventilation, highlighting the impaired ability to effectively ventilate the patient and maintain optimal gas exchange. This reduction in tidal volume further underscores the importance of considering the mechanical impact of laryngeal masses in airway management.

Previous reports indicate that the insertion of the supraglottic airway can cause the epiglottis to fold, which may lead to glottic obstruction [9]. Folding of the epiglottis is a well-known risk factor for difficulties in ventilation [10]. As shown in Figure 2, the presence of an epiglottic mass, particularly in large cases, may create conditions that lead to epiglottic folding due to deep insertion of the supraglottic airway. Consequently, despite achieving easy face mask ventilation after muscle relaxation, ventilation may become difficult following the insertion of the i-gel®.

Previous reported risk factors for ventilation failure following supraglottic airway insertion include male gender, age over 45, short thyromental distance, and limited neck extension from the retrospective review of 14,480 patients aged ≥ 18 years who underwent general anesthesia [11]. Based on the results of this study, large epiglottic masses could also be a risk factor for ventilation difficulties following supraglottic airway insertion. Furthermore, because epiglottic masses are generally asymptomatic, they can be difficult to detect during preoperative examinations. Therefore, anesthesiologists should acknowledge that ventilation failure may occur unexpectedly due to the presence of an epiglottic mass.

The presence of an epiglottic mass can sometimes hinder the insertion of the laryngoscope into the epiglottic vallecula, potentially obstructing the induction of general anesthesia. However, this may depend on the size and location of the mass. There are reports of successful intubation using the McGRATH® in a case of an epiglottic cyst [12] and the Airway Scope in a case of congenital lingual thyroglossal duct cyst [13]. Since laryngeal masses can sometimes be malignant [14], it could be important to minimize the risk of rupture, especially because laryngoscopes may increase this risk as they lift the epiglottis. Furthermore, fiberoptic bronchoscopy-guided intubation may be challenging when advancing the endotracheal tube due to the potential for blind advancement by blood or secretions in the airway [15]. Therefore, the choice of which device to use in anesthetic management should be determined on a case-by-case basis.

The limitations of this study include the use of an airway management simulator, which may not precisely reproduce the anatomy of an actual larynx. In addition, air leaks occurred around the cuff of the i-gel® at inspiratory pressures above 25 cmH₂O, making it impossible to measure tidal volume accurately at higher pressures. While i-gel® was used as the supraglottic airway in this study, results may vary with other supraglottic airways.

## Conclusions

To date, the use of supraglottic airways for managing epiglottic masses in airway management remains uncertain. Epiglottic masses are often asymptomatic, making them difficult to detect preoperatively. As a result, anesthesiologists may face unexpected ventilation challenges. While supraglottic airways may help secure the airway, they might not resolve ventilation failure, especially with larger or heavier masses. Nonetheless, they can still be a useful option in some cases.

## References

[1] Tresley J, Saraf-Lavi E, Kryvenko ON, Sargi Z (2015). Epiglottic masses identified on CT imaging: a case report and review of the broad differential diagnosis. Neuroradiol J.

[2] Varathan V, Mat Baki M, Kalimuthu S (2023). A case report of an unusual presentation of epiglottic cyst causing airway obstruction in an adult. Cureus.

[3] Zhang R, Jiang X, Feng J (2023). Difficult endotracheal intubation due to a large epiglottic cyst: a case report. Medicine (Baltimore).

[4] Apfelbaum JL, Hagberg CA, Connis RT (2022). 2022 American Society of Anesthesiologists practice guidelines for management of the difficult airway. Anesthesiology.

[5] Herrera K, Tufail B, Osborn I (2024). Innovative (and safe) techniques with supraglottic airways. Int Anesthesiol Clin.

[6] ApIvor D (1972). Epiglottic cysts. Anaesthesia.

[7] Shaik F, Chowdary K (2016). A clinical study of laryngeal cysts. Int J Phonosurg Laryngol.

[8] Jannu A, Shekar A, Balakrishna R, Sudarshan H, Veena GC, Bhuvaneshwari S (2017). Advantages, disadvantages, indications, contraindications and surgical technique of laryngeal airway mask. Arch Craniofac Surg.

[9] van Zundert AA, Wyssusek KH (2018). Epiglottis folding double with supraglottic airway devices. Br J Anaesth.

[10] Suzuki A, Yamada N, Seki K, Hotta K (2024). Classification of epiglottal displacement. Anesthesiology.

[11] Saito T, Liu W, Chew ST, Ti LK (2015). Incidence of and risk factors for difficult ventilation via a supraglottic airway device in a population of 14,480 patients from South-East Asia. Anaesthesia.

[12] Takahashi K, Saima S, Arai T, Okuda Y (2016). Utility of McGRATH MAC for a patient with a large epiglottic cyst [Article in Japanese]. Masui Jpn J Anesthesiol.

[13] Kohashi Y, Yamamoto T, Igarashi M, Nishimaki H (2022). Unexpected difficult airway due to an undiagnosed congenital lingual thyroglossal duct cyst in a neonate without stridor: A case report. Clin Case Rep.

[14] Salerno G, Mignogna C, Cavaliere M, D'Angelo L, Galli V (2007). Oncocytic cyst of the larynx: an unusual occurrence. Acta Otorhinolaryngol Ital.

[15] Heidegger T, Gerig HJ, Ulrich B, Schnider TW (2003). Structure and process quality illustrated by fibreoptic intubation: analysis of 1612 cases. Anaesthesia.

